# A Structured Protocol Model of Depression Care versus Clinical Acumen: A Cluster Randomized Trial of the Effects on Depression Screening, Diagnostic Evaluation, and Treatment Uptake in Ugandan HIV Clinics

**DOI:** 10.1371/journal.pone.0153132

**Published:** 2016-05-11

**Authors:** Glenn J. Wagner, Victoria Ngo, Prodyumna Goutam, Peter Glick, Seggane Musisi, Dickens Akena

**Affiliations:** 1 RAND Corporation, Santa Monica, California, United States of America; 2 Department of Psychiatry, Makerere University, Kampala, Uganda; Nagoya University Graduate School of Medicine, JAPAN

## Abstract

Depression is common among people living with HIV, and it has consequences for both HIV prevention and treatment response, yet depression treatment is rarely integrated into HIV care in sub-Saharan Africa, partly due to the paucity of mental health professionals. We conducted a cluster randomized controlled trial of two task-shifting models to facilitating depression care delivered by medical providers: one that utilized a structured protocol, and one that relied on clinical acumen, in 10 HIV clinics in Uganda. Both models started with routine depression screening of all clients at triage using the 2-item Patient Health Questionnaire (PHQ-2), from which we enrolled 1252 clients (640 at structured protocol clinics, 612 at clinical acumen clinics) who had screened positive over 12 months. We compared the two models on (1) proportion of all client participants, and those clinically depressed (based on survey-administered 9-item PHQ-9>9), who received post-screening evaluation for depression using the PHQ-9; and (2) proportion of clinically depressed who were prescribed antidepressant therapy. Linear probability regression analyses were conducted using a wild cluster bootstrap to control for clustering; patient characteristics, clinic size and time fixed effects were included as covariates. Among all client participants, those in the structured protocol arm were far more likely to have received further evaluation by a medical provider using the PHQ-9 (84% vs. 49%; beta = .33; p = .01). Among the clinically depressed clients (n = 369), the advantage of the structured protocol model over clinical acumen was not statistically significant with regard to PHQ-9 depression evaluation (93% vs. 68%; beta = .21; p = .14) or prescription of antidepressants (69% vs. 58%; beta = .10; p = .50), in part because only 30% of clients who screened positive were clinically depressed. These findings reveal that in both models depression care practices were widely adopted by providers, and depression care reached most depressed clients. The structured protocol model is advantageous for ensuring that positively screened clients receive a depression evaluation, but the two models performed equally well in ensuring the treatment of depressed clients in the context of strong supervision support.

***Trial Registration*:** ClinicalTrials.gov NCT02056106

## Introduction

Depression is common among people living with HIV (PLWHIV) in sub-Saharan Africa (SSA), with rates of clinical depression ranging from 10–20%, and an additional 20–30% having elevated depressive symptoms [1,2]. Depression can have significant consequences for HIV disease progression [3] and treatment response [4], as well as ability to use condoms consistently [5]. Yet despite the availability of effective treatments for depression in PLWHIV [6], including antidepressants [7], and the benefits of depression treatment for HIV antiretroviral therapy (ART) use, adherence and outcomes [8,9], depression treatment is rarely integrated into HIV care programs across SSA [2].

A severe shortage of mental health professionals is a key barrier to depression care in Uganda and the larger region [10]. Task-shifting models of care, in which lower trained cadres take on duties traditionally performed by more highly trained providers, present a solution to deficits in highly trained health care providers [11]. Collaborative care models of depression treatment, which combine task-shifting, use of a structured, algorithm-based protocol, and supervision by a specialist, have been implemented successfully with non-HIV clients in the U.S. [12,13] and resource constrained settings such as India [14]. A few studies have also demonstrated the efficacy of this approach with HIV clients in the U.S. [15, 16], but we are aware of only a small pilot study of its use with this population in Africa [17]. Although task-shifting is necessary in low resource settings, differing models for task-shifting have neither been compared against each other, nor have certain components of task-shifting been tested to identify and guide the process of integrating depression care into HIV care settings in SSA.

***INDEPTH*** (***IN****tegration of* ***DEP****ression* ***T****reatment in* ***H****IV care*) ***Uganda*** is a cluster randomized trial that compared two task-shifting implementation models of depression care: a *structured protocol model* in which care was provided largely by trained nurses who acted as depression care managers, and a model that relied on the *clinical acumen* of trained primary care providers (most, but not all, of whom were nurses as well). Providers in both models received the same workshop training on how to evaluate and treat depression, and monthly on-site supervision, and both models relied on trained expert clients to administer a brief depression screening at triage; however, in the structured protocol model this depression screen was to be followed up by further evaluation, diagnosis and treatment by trained nurses using a systematic, structured protocol, while in the clinical acumen model the trained primary care providers used their clinical discretion regarding whether to pursue further evaluation and treatment. Both models go beyond usual care, which has no depression screening and relies solely on primary care providers who have relatively no depression care training to identify and treat depressed clients, or refer to external specialists, resulting in depression rarely being assessed or treated. The models also go beyond the common Ministry of Health approach in low resource settings of delivering initial provider training for new services but no mentorship or ongoing supervision, which may be critical for successful provider adoption and maintenance of new care processes [18]. Furthermore, the structured protocol model may be more resource intensive if the protocol triggers more service provision, therefore the comparison of it with the clinical acumen of trained, supervised providers will inform policy decisions on how to integrate depression care into HIV clinics.

The RE-AIM (Reach, Effectiveness, Adoption, Implementation, Maintenance) implementation framework stipulates that for an intervention to have an impact on health at the population level it must be *adopted* by providers, *reach* a large proportion of the client population, be *implemented* with fidelity, *effectively* improve outcomes, and be *maintained* post study [19].

While the objectives of INDEPTH Uganda are to evaluate the two models of depression care on each of these domains, for this paper we evaluated the implementation of these two task-shifting models in terms of the adoption of screening, evaluation and treatment practices by providers and the reach of these practices to clients at high risk for depression. We compared the structured protocol and clinical acumen models on the implementation of the three initial steps of depression treatment initiation: (1) depression screening (proportion of clients visiting the clinic who are screened for depression); (2) depression diagnosis (proportion of clients who screen positive for potential depression who are further evaluated for depression diagnosis; and (3) depression treatment (proportion of clients who meet criteria for depression who are prescribed antidepressant treatment). We hypothesized that the models would not differ with regard to depression screening as this component was identical in both models and was to be implemented uniformly, but that the structured protocol model would result in a greater proportion of clients receiving depression evaluation and antidepressant treatment as these processes are more complex and the structured protocol outlines clear decision points and steps to be taken, which may be key to facilitating depression care among providers with minimal training. We also compared trajectories of implementation change over time in order to further assess the adoption and maintenance of depression care processes by the providers.

## Methods

### Study Design

INDEPTH-Uganda is a comparative efficacy trial that compares two active task-shifting implementation models for integrating antidepressant treatment into HIV care within 10 health care facilities in Uganda. Using a cluster randomization, 5 clinics were assigned to implement a structured protocol model, and 5 others relied on the clinical acumen of trained providers. A usual care (no-intervention) control was not included in the design, because depression care was by and large nonexistent in the study setting, rendering comparisons with usual care as uninformative. The selection of 10 sites (clusters) was based on feasibility given the funding level of the grant; 150 patients per site, an assumed 30% antidepressant uptake rate in the clinical acumen model, and an intracluster correlation coefficient of .16 (the median found in a study of health outcomes in 8 developing countries) [20], provides the power (α = .05, 80% power; one sided test) to detect an increase of 28% in antidepressant uptake rates between the two depression care models.

The models were implemented over 24 months starting in January 2013. To evaluate the models, data were collected from documentation mechanisms integrated into routine care, and a cohort comprised of adult clients who screened positive for potential depression at triage. A more detailed description of the study protocol has been published elsewhere [21], and the trial has been registered with the National Institutes of Health sponsored clinical trials registry (NCT02056106); note that the trial was registered after the start of enrollment because the investigators were not familiar with the requirements for trial registration. The authors confirm that all ongoing and related trials for the study interventions are registered. The study protocol was approved by Institutional Review Boards at the RAND Corporation and Makerere University School of Medicine Ethical Research Committee, as of July 12, 2012.

### Study Setting

The study was conducted in collaboration with Mildmay Uganda, a non-government organization that provides holistic outpatient HIV care at its own clinics, as well as technical assistance in HIV care to healthcare facilities across Uganda, including the study sites. Of the ten healthcare facilities participating in the study, eight are run by the Ministry of Health and two are private, faith-based, not-for-profit healthcare facilities; two are district hospitals and the others are designated as health centre III or IV facilities by the Uganda Ministry of Health, and are located in the districts of Mpigi, Mityana, Luweero, and Wakiso. Each facility is a hospital that operates an HIV clinic on specific designated days of the week, and it is in these clinics that depression care was integrated as part of this study. The six larger clinics operate 2–3 days per week and generally have one clinical or medical officer and 3 to 5 nurses to provide primary HIV care to a clientele ranging from 1500–3000 clients. The four smaller clinics operate one day per week and are manned by one clinical or medical officer and 2–3 nurses; client base ranges from 350–1000.

Nurses serve as primary care providers (along with the clinical/medical officers) and manage the prescription and monitoring of ART and other common HIV medications at all clinics. Complex conditions or complications that arise are typically managed by the clinical/medical officer. All clinics have expert clients (volunteer, experienced HIV clients who display exemplary HIV care adherence and retention, many of whom are also village health team workers) who volunteer to take on tasks such as triage assessments, and filing and retrieving charts. No psychiatric or depression care services were being provided at these clinics as part of usual care prior to the study; as part of usual care, clients who developed significant psychiatric symptoms were typically referred to the nearest district or regional hospital for care.

### Randomization and Masking

To ensure the two study arms were balanced on clinic size, separate randomization drawings were conducted within the 6 larger clinics and the 4 small clinics. At a meeting attended by representatives from all study sites, names of the sites were placed on pieces of paper that were folded and placed into a hat, from which one of the study investigators drew the assignments to the two study arms in alternating order. There was no masking to model assignment in this study.

### Longitudinal Client Cohort

This cohort was enrolled between January and December of 2013, with data collection completed by December 2014. Clients who screened positive for potential depression on the 2-item Patient Health Questionnaire [22] (PHQ-2 ≥ 3) at triage were eligible candidates for enrollment into the cohort if they were at least 18 years of age, medically stable (not about to start [or recently started] ART or treatment for an acute opportunistic infection), and the research coordinator confirmed their depression status after re-administering the PHQ-2. All clients who screened positive for potential depression on the days of recruitment (one day per week at each site) were informed of the study and asked to provide written informed consent. At sites that were open more than one day per week, clients could have screened positive, been diagnosed and treated for depression on non-recruitment days, but than enrolled at a later clinic visit that coincided with a recruitment day if they continued to screen positive for depression. Records on refusals were not kept, but research coordinators indicate that the vast majority (> 95%) of eligible clients agreed to participate. Participants were followed for 12 months with assessments at baseline and months 6 and 12. Participants received 10,000 Ush (~$4 USD) for completing each assessment.

### Task-Shifting Depression Care Models

This paper focuses on comparing the two models of depression care on implementation outcomes related to depression screening, diagnosis and treatment prescription; therefore, we describe in detail below only the model components related to these care processes. A detailed description of each component of both models has been published elsewhere [21] both models solely focus on the use of antidepressants to treat depression. Implementation of the models began after a one-day training workshop for nurses and clinical/medical officers, on-site training with expert clients, and on-site training and mentorship from the supervising psychiatrist and research coordinators that continued throughout the two-year implementation.

#### Structured protocol model

Routine depression screening: All adult clients were to be screened for depression at each clinic visit using the PHQ-2 administered at the triage station by trained expert clients. The PHQ-2 assesses depressed mood and loss of interest, with scores ≥ 3 (possible range: 0–6) representing potential depression. PHQ-2 scores were noted in the client’s clinic book and relayed to the nurse for further evaluation.

Depression diagnosis: Clients who screened positive for potential depression and were deemed medically stable were to be further evaluated using the full 9-item PHQ-9, which has been used successfully to assess depression in sub-Saharan Africa.[23] Each item of the PHQ-9 corresponds to the 9 symptoms assessed in the depression module of the Diagnostic Statistical Manual of Mental Disorders (DSM-V) [24]. Clients who scored greater than 9 on the PHQ-9 (possible range: 0 to 27), which has been shown to correspond highly with Major Depression as determined by a diagnostic interview [22], were to be further assessed using the criteria for Major Depression in the Mini International Neuropsychiatric Interview [25] (MINI; 5 symptoms scored as 3 on the PHQ-9, at least one of which is depressed mood or loss of interest, plus functional impairment). Nurses further assessed antidepressant eligibility using the MINI screening items for bipolar disorder, psychosis and substance abuse, as well as any medical contraindications; for clients with these conditions, the supervising psychiatrist was consulted to determine appropriate treatment.

Prescription of antidepressants: If eligibility for antidepressant therapy was confirmed, the nurse educated the client about depression and what to expect from treatment, then prescribed either fluoxetine or imipramine depending on the client’s presenting symptoms and psychiatric history.

Supervision: After the initial on-site supervision and mentorship that took place weekly until the nurses displayed sufficient competency and confidence (4 to 6 weeks, depending on the site), supervision visits to each site occurred monthly. Supervision was conducted in one-on-one sessions between the supervisor and each nurse, as well as group meetings with all clinic staff involved with implementation of depression care at the site. During individual supervision, new and problematic or non-responding cases were presented and treatment plans discussed. Clinical notes from the Depression Treatment Registry were used in case and chart reviews. At each supervision visit, supervisors reviewed the charts of all patients prescribed antidepressants within the past month, as well as 10 randomly selected charts of patients receiving ongoing antidepressant treatment monitoring. The charts were reviewed for whether diagnosis, symptoms and side effect assessment, and dosing were appropriately performed, and if the patient returned for follow-up visits. The group staff meetings provided an opportunity for the clinic staff to work as a team to trouble-shoot any challenges, share experiences and provide peer support. Furthermore, the supervisor was available by phone at all times for emergency or suicide crisis consultations.

#### Clinical acumen model

Like the structured protocol model, the clinical acumen model also includes routine screening of all adult clients with the PHQ-2 by expert clients at triage, the information from which was relayed to the primary care provider; however, this model relies on the clinical judgment of the primary care provider to decide whether to further evaluate and treat clients who screen positive for possible depression (PHQ-2 ≥ 3), as opposed to following a structured protocol. Yet when providers chose to evaluate or treat, they were trained to use the same methods of diagnosis and treatment as those in the structured protocol model. Aside from not using a structured protocol to determine when to diagnose and treat, the other key difference between the two models is one aspect of supervision; in the structured protocol model, supervisors used data provided by the research coordinators to monitor how well the providers were adhering to the structured protocol and provided intermittent feedback to encourage compliance with the protocol; in contrast, supervision in the clinical acumen arm focused mostly on clients who were being treated.

### Outcome Measures

#### (1) Depression screening

*Proportion of clients visiting the clinic who were screened for depression using the PHQ-2*. This parameter was assessed by abstracting data from the Triage Book, which the triage station at each site uses to list the clients who attend each day of the clinic, along with their age, sex, vital signs, presenting problems and PHQ-2 score.

#### (2) Depression diagnostic evaluation

*Proportion of clients who screened positive for potential depression who were further evaluated for depression diagnosis*. This parameter was assessed using data from the client research cohort, which was comprised of medically stable clients who had screened positive for potential depression at triage and thus should have received further evaluation for depression from their providers during the same clinic visit. PHQ-9 and MINI data from clinic charts were used to assess whether a depression diagnostic evaluation took place. Furthermore, using data from the subgroup of clients in the research cohort who met criteria for clinical depression (baseline survey PHQ-9>9), we examined the *proportion of depressed clients who were evaluated for depression diagnosis* by providers.

#### (3) Antidepressant treatment

*Proportion of depressed clients who were prescribed antidepressant treatment*. To assess this parameter, data from the client research cohort were used to determine the pool of medically stable clients with clinical depression (baseline survey PHQ-9>9), and data regarding prescription of antidepressants to these clients were abstracted from the clinic’s Depression Treatment Registry. These registry books were installed at each site for providers to document clients who were prescribed antidepressants, their depression diagnosis, and data collected at monitoring visits (PHQ-9 score, side effects, medication dose).

### Measures of Client Characteristics

Demographics included in the analysis included gender, age, and education (whether or not the participant had any secondary education). CD4 count at study entry and whether or not the participant was on antiretroviral therapy were abstracted from the participant’s clinical chart. Physical health functioning was measured with the 6-item subscale of the Uganda-adapted Medical Outcomes Study HIV Health Survey [26]. The survey assessment also included measures of economic and psychosocial functioning, and HIV disease management, but we are not including details of these measures here as they were not included in the analyses.

### Analysis

Descriptive statistics were used to describe the number and proportion of clients who were screened, received a diagnostic evaluation, and treated with antidepressants. For proportion of clients screened, data are at the clinic level and we compared the two treatment models using simple bivariate (two-tailed) statistics. Multivariate analysis of client level data from the research cohort was used to compare the two models on the proportions of clients that received a diagnostic evaluation and antidepressant treatment. The regression models to estimate these two outcomes included baseline covariates to adjust for basic demographics (age, sex, education) and variables that may be associated with the dependent variables (ART status, CD4 count [which was transformed using a division of 100 to improve the interpretability of the beta coefficient], physical health functioning, depression severity [PHQ-9], clinic size). The models also included time fixed effects to control for differences in client and clinic characteristics over the first 12 months of implementation (the time period in which clients were enrolled in the research cohort); data from months 1 and 2 (January and February) were combined, as were the data from months 11 and 12 (November and December), because participant enrollment started near the end of month 1 and ended mid month 12 (just prior to the holiday break for the clinics).

Since the diagnostic evaluation and treatment outcomes are modeled using client level data rather than clinic level means, we needed to account for the clustering of the client data by clinic. This is usually done by estimating the intracluster correlation coefficient (ICC), which measures the variation between clusters as a share of total variation (between and within clusters). However, when the number of clusters is very small, additional methods to estimate the ICC, derived standard errors and p-values have been shown to be unreliable [27, 28]. An increasingly common approach to get around this problem is a bootstrapping approach known as the wild cluster bootstrap [28], which we apply using a program developed for STATA software [29]. This technique does not generate meaningful standard error statistics, so we only report beta coefficients and p values. The program is only available for linear regressions, so we estimated a linear probability model for the outcomes, but supplemented this with robustness tests to compare the estimated coefficients from the linear models with average marginal effects from logit regression models. One limitation of the wild bootstrap is that it does not extend to cases of multiple level clustering. In the present case, we have clustering at the clinic level and the client level, since the analysis uses repeated observations per person. However, accounting for clinic-level clustering in a study with a small number of clusters is expected to be far more important in terms of avoiding biased inferences. Further, by not also accounting for within-person correlations, our findings are more conservative, since use of repeated observations reduces variance.

## Results

**Fig 1:**
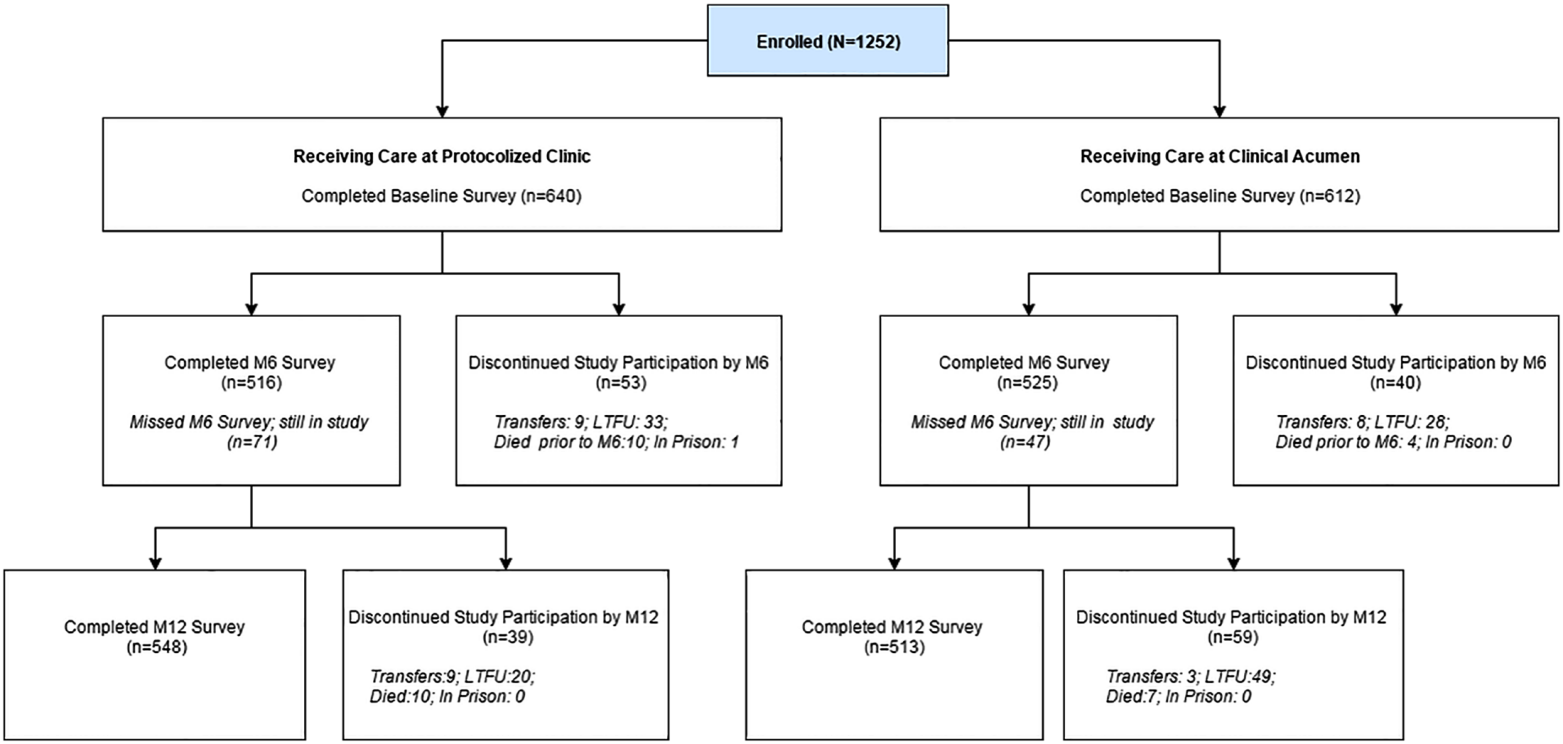
The CONSORT Flowchart.

A sample of 1252 clients (range of 82 to 182 clients at each site) enrolled in the longitudinal cohort, including 640 and 612 clients from sites in the structured protocol and clinical acumen arms, respectively. Participant characteristics are listed in Table 1; there were no significant differences between those in the clinical acumen arm and those in the structured protocol arm. Fig 1 depicts the flow of participants in both arms through the study protocol.

**Table 1:** Participant Characteristics for the Total Sample and by Study Arm. SD = standard deviation.

	Total Sample (N = 1252)	Structured Protocol (N = 640)	Clinical Acumen (N = 612)
Mean (SD) PHQ-9 total score	8.2 (4.3)	8.2 (4.2)	8.2 (4.4)
PHQ-9 = 0–9	70.5%	70.2%	70.7%
PHQ-9 = 10–14	19.5%	20.2%	18.7%
PHQ-9 = 15–27	10.1%	9.6%	10.6%
Secondary education (%)	18.6%	17.3%	19.9%
Mean (SD) Age	40.0 (11.2)	40.6 (11.0)	39.3 (11.3)
Female (%)	76.8%	74.7%	78.9%
Mean (SD) CD4 (cells/mm^3^)	430 (270)	431 (274)	429 (266)
Mean (SD) physical health functioning	79.1 (22.7)	77.9 (23.6)	80.4 (21.7)
On antiretroviral therapy (%)	73.7%	72.7%	74.8%
Mean (SD) months since HIV diagnosis	44.6 (40.8)	46.4 (37.1)	42.4 (44.4)

### Depression Screening

The mean number of adult clients seen each clinic day across all sites in the structured protocol arm was 69.1 (SD = 33.5; range: 35.6–97.8 across sites), which was more than the 58.0 (SD = 30.6; range: 29.4–90.3) clients seen in the clinical acumen arm (t = 7.35, df = 1641, p < .0001). In the structured protocol arm, an average of 76.3% (SD = 20.1; range: 68.9–91.7% across sites) of adult clients was screened at triage with the PHQ-2, which was slightly lower than the 80.0% (SD = 18.4; range: 59.9–90.6%) in the clinical acumen arm (t = -4.09, df = 1662, p = .038). The rate of screening was tracked monthly at each site and remained relatively stable over the course of the first 12 months of implementation in the structured protocol arm (80.4% in January/February, 74.2% in November/December) and the clinical acumen arm (74.7% in January/February, 79.8% in November/December). Among those screened at sites in the structured protocol arm, 8.2% (SD = 11.02; range: 5.3–14.9% across sites) screened positive for possible depression (PHQ-2 ≥ 3), which was equivalent to the 8.0% (SD = 9.06; range: 2.7–14.9%) found in the clinical acumen arm (t = 0.52, df = 1552, p = .167).

### Depression Diagnostic Evaluation

At sites in the structured protocol arm, 83.6% (535/640) of clients enrolled in the research cohort (all of whom were medically stable, had screened positive for potential depression, and thus should have received further depression evaluation) were further assessed for depression using the PHQ-9 and MINI, compared to 48.9% (299/612) in the clinical acumen arm (Chi Square = 169.7, df = 1, p < .0001). Multivariate regression analysis revealed that being in the structured protocol arm was associated with a greater likelihood of having a diagnostic evaluation (beta = .330; p = .014), as was greater depressive symptoms as represented by a baseline survey PHQ-9 score of 10–14 (compared to scores < 9) (beta = .155, p = .034); a PHQ-9 score of 15 or greater was marginally associated (beta = .187, p = .082) with having a diagnostic evaluation (see Table 2). There was considerable variation across sites within each depression care model on proportion of participants who received a diagnostic evaluation, from 34.1% to 72.3% in the clinical acumen arm, and 62.1% to 96.6% in the structured protocol arm.

**Table 2:** Multivariate regression analyses comparing structured protocol model of depression care vs. clinical acumen on implementing depression diagnostic evaluations and prescription of antidepressants.

	Diagnostic Evaluation (Whole Sample)	Diagnostic Evaluation (Clinically Depressed)	Prescription of Antidepressants
	Beta (p value)	Beta (p value)	Beta (p value)
Structured protocol model	0.33 (0.014)	0.211 (0.124)	0.084 (0.589)
Baseline depression severity			
PHQ-9 = 0 to 9	Referent	N/A	N/A
PHQ-9 = 10–14	0.155 (0.034)	Referent	Referent
PHQ-9 = 15–27	0.187 (0.082)	0.026 (0.551)	0.188 (0.002)
Secondary education	0.022 (0.515)	0.006 (0.865)	0.009 (0.903)
Age	-0.001 (0.577)	-0.0003 (0.881)	0.0005 (0.813)
Female	0.024 (0.302)	0.054 (0.286)	0.121 (0.178)
CD4 cell count	0.01 (0.198)	0.01 (0.206)	0.02 (0.056)
Physical health functioning	-0.001 (0.184)	0.002 (0.028)	0.002 (0.066)
Large clinic	-0.101 (0.294)	-0.162 (0.190)	-0.145 (0.384)
On HIV antiretroviral therapy	-0.015 (0.462)	-0.037 (0.458)	-0.003 (0.983)
Month of enrollment			
January/February	Referent	Referent	Referent
March	0.044 (0.615)	-0.146 (0.292)	-0.016 (0.881)
April	0.161 (0.042)	0.041 (0.631)	0.030 (0.823)
May	0.118 (0.158)	0.029 (0.713)	0.012 (0.867)
June	0.081 (0.286)	0.002 (0.883)	0.056 (0.715)
July	0.048 (0.523)	-0.250 (0.336)	-0.159 (0.424)
August	0.077 (0.478)	-0.008 (1.000)	-0.033 (0.895)
September	-0.018 (0.833)	0.023 (0.785)	-0.004 (1.000)
October	-0.037 (0.601)	-0.016 (0.893)	-0.018 (0.977)
November/December	-0.172 (0.190)	-0.108 (0.474)	0.007 (0.965)
Constant	0.528	0.641	0.263
Observations	1,249	369	369
R-squared	0.233	0.225	0.126

**Fig 2:**
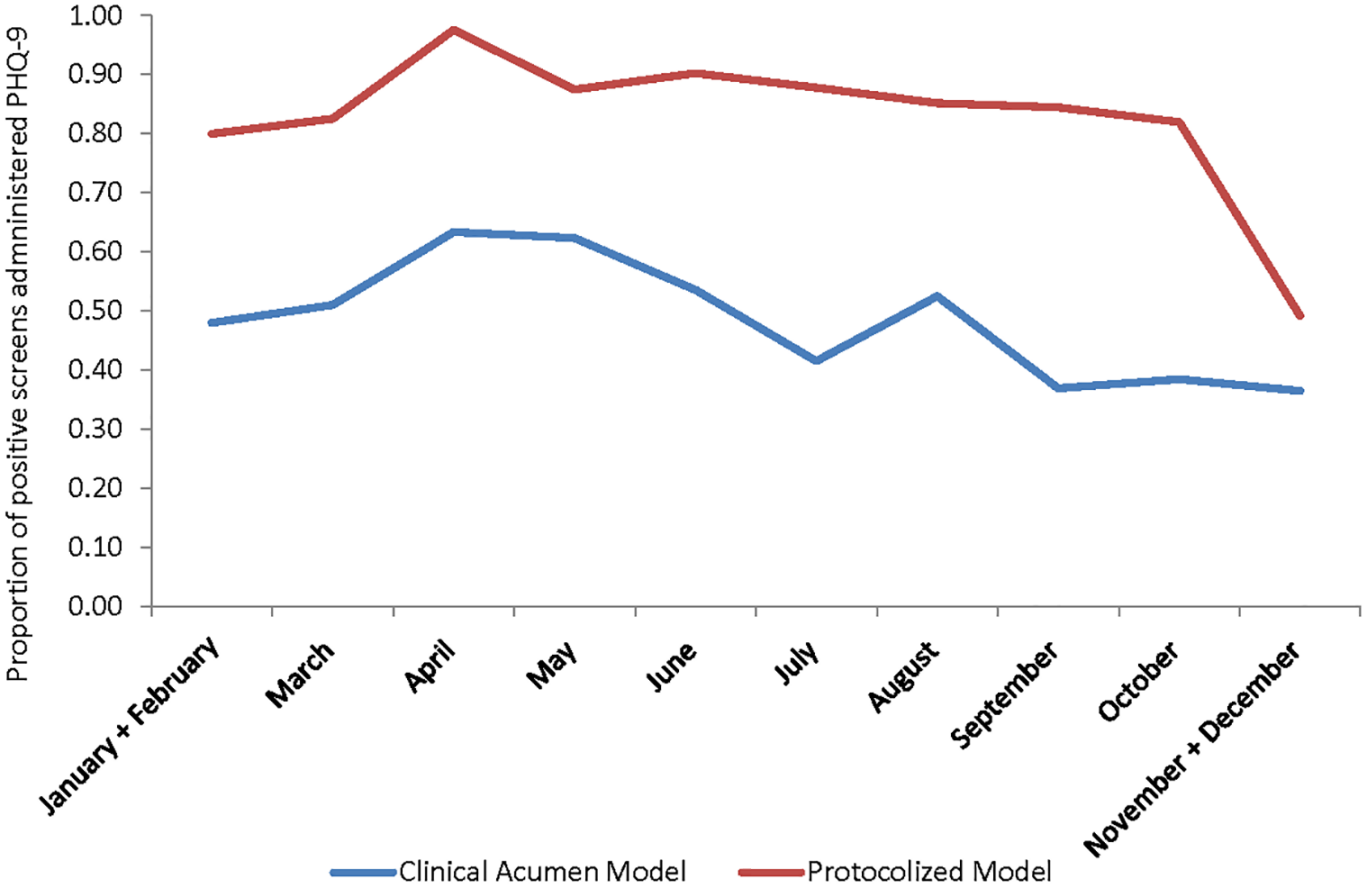
Proportion of All Participants Administered Depression Diagnostic Evaluation by Month of Enrollment.

Fig 2 shows the proportion of all participants who received a diagnostic evaluation each month over the course of the first 12 months of implementation for each model; the line graph shows that the structured protocol model remained superior throughout the duration of the 12 months. The multivariate regression analysis, which included fixed effect coefficients for each month (with combined data from January and February as the reference), revealed little variance over time within each model (see Table 2); performance in April was the only month that was significantly different (improved) from that of January/February, which coincided with when on-site supervision shifted from weekly to monthly. Performance in the structured protocol arm remained particularly stable throughout, but there was a decline in the last month, which may be related to the impending holiday season and its effects on clinic operations and staffing.

A total of 369 clients (29.5%) were clinically depressed (based on baseline survey PHQ-9>9), including 190 in the structured protocol arm and 179 in the clinical acumen arm. Among the clinically depressed, 92.6% (176/190) received a diagnostic evaluation by providers in the structured protocol arm, compared to 68.2% (122/179) in the clinical acumen arm (Chi Square = 35.5, df = 1, p < .0001). Multivariate analysis revealed that this advantage of the structured protocol model was not statistically significant (beta = .211, p = .124); the only variable associated with having a depression evaluation was better physical functioning (beta = .002, p = .028) (see Table 2).

The robustness tests with logit regression models produced similar results as those described above, with the same variables being significantly associated with diagnostic evaluation in both the whole sample and the clinically depressed subgroup.

### Depression Treatment

**Fig 3:**
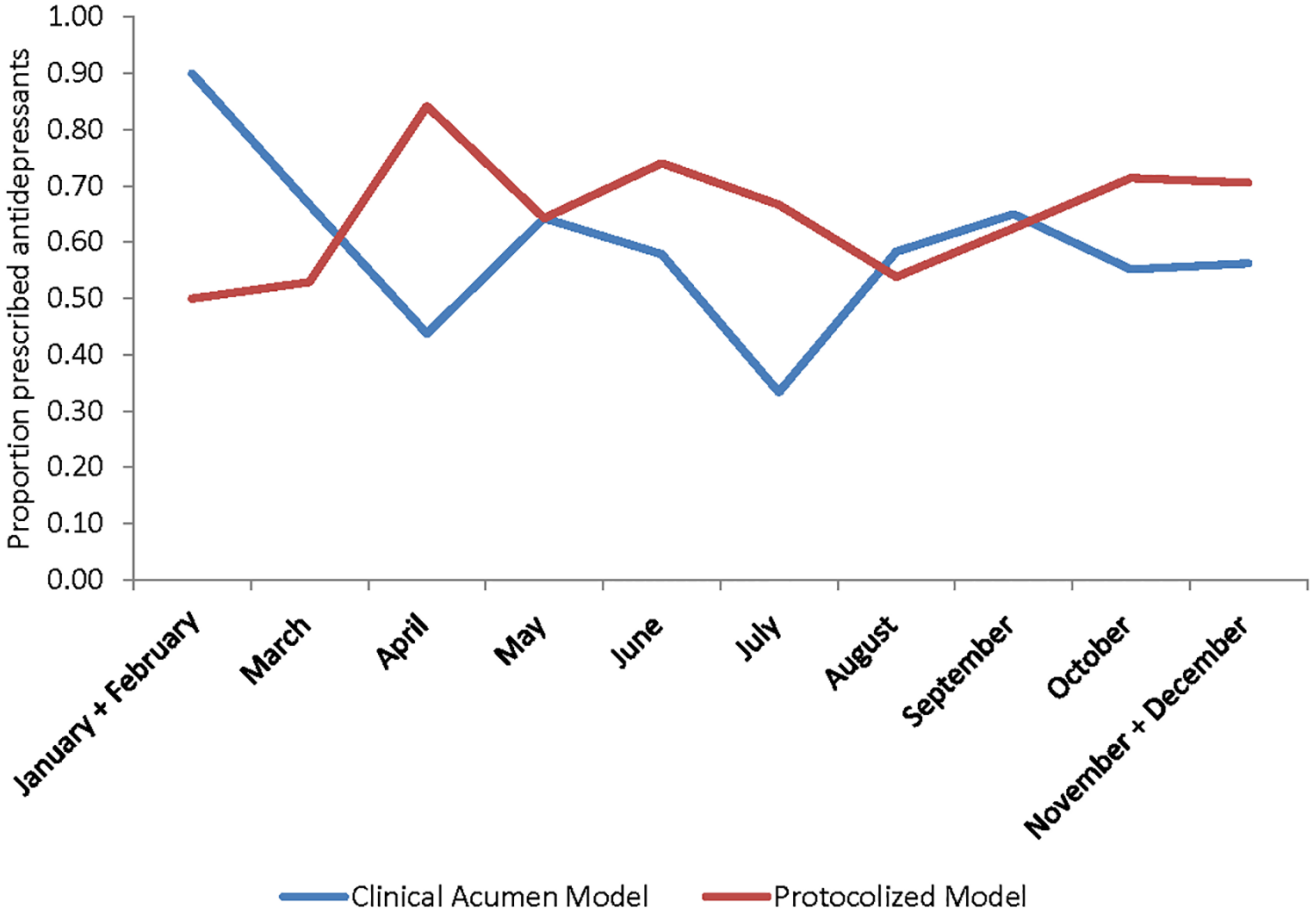
Proportion of Clinically Depressed Participants Prescribed Antidepressants by Month of Enrollment.

Of the 176 clinically depressed participants in the structured protocol arm who received a diagnostic evaluation from their provider, 134 (76.1%) were diagnosed with major depression based on the PHQ-9 and MINI, compared to 103 (84.4%) of the 122 clinically depressed participants in the clinical acumen arm who received a diagnostic evaluation (Chi Square = 3.0, df = 1, p = .081). Of those who were diagnosed with major depression by their provider, 97.8% (131/134) received antidepressant therapy in the structured protocol arm, and 100% (103/103) in the clinical acumen arm. Among all clinically depressed participants (n = 369), this represents a treatment rate of 68.9% (131/190) in the structured protocol arm versus 57.5% (103/179) in the clinical acumen arm (Chi Square = 5.2, df = 1, p = .023). The variation between sites within each arm ranged from 25.0% to 80.8% in the clinical acumen arm, and 41.0% to 80.6% in the structured protocol arm. In the multivariate regression analysis, the depression care model was not significantly associated with treatment uptake (beta = .084, p = .589); the only significant correlate was severe depression symptoms (PHQ-9 score of 15–27; beta = .188, p = .002), while higher CD4 count (beta = .0002, p = .056) and better physical health functioning (beta = .002; p = .066) were marginally related to treatment uptake (see Table 2). Rates of uptake were relatively stable over time as illustrated in Fig 3, and supported by the regression model (see Table 2) in which none of the monthly rates differed significantly from the reference (combined rate of January and February). Similar results were observed in the robustness test with a logit regression model.

## Discussion

This study is one of few to publish data on the implementation of depression care for PLWHIV in SSA, and perhaps the first to compare the implementation of two task-shifting approaches in this context. Together with findings from our prior analysis, which demonstrated accurate PHQ-2 and PHQ-9 depression evaluations by expert clients and medical providers, respectively [30], these results provide evidence that routine brief depression screening, diagnostic evaluation, and prescription of antidepressant treatment can be implemented well by HIV clinic staff with appropriate training and ongoing supervision and mentorship. Both models of depression care resulted in most clinically depressed clients being diagnosed and treated at steady rates over one year, demonstrating good adoption of depression care practices by providers and staff, as well as good reach of depression care to depressed clients. The structured protocol model of depression care showed some evidence of being superior in ensuring a diagnostic evaluation, but this advantage was attenuated among clinically depressed clients.

In both the clinical acumen and structured protocol arms, the vast majority of adult clients were screened for depression each clinic day and consistently over the course of the study, demonstrating that routine depression screening by trained layman is feasible. In both models, just under 10% of clients screened positive for possible depression—a rate that is low compared to other studies of PLWHIV in SSA which have reported rates of elevated depressive symptoms in the range of 30–50% [1,2], but consistent with our prior research with HIV clients in Uganda who have been receiving HIV care for some time and not newly diagnosed or new to care [31]. Also, many of these clients were on ART, which we have found to be associated with reduced depression in this setting [31].

In the structured protocol arm, the vast majority (84%) of medically stable clients who screened positive on the PHQ-2 at triage received a diagnostic evaluation by a trained nurse, compared to half of such clients in the clinical acumen arm, and performance was consistent over time. This finding highlights the value of a structured protocol for ensuring the implementation of next steps in depression care, compared to reliance on provider judgment, even though all providers in both arms receive the PHQ-2 screening information. However, this advantage of the structured protocol model was less evident among the subgroup of clinically depressed participants (93% vs. 68%). One possible explanation for this finding is the inaccuracy of the PHQ-2 screen, as less than one third of clients who screened positive on the PHQ-2 were classified as clinically depressed according to the survey PHQ-9. This low positive predictive value of the PHQ-2 is equivalent to what was found in another study of the PHQ-2 conducted in SSA [23]; this other study also reported very high sensitivity and specificity for depression classifications on the PHQ-2 (scores ≥ 3) compared to the PHQ-9 (scores > 9), but a scale can have low positive predictive value despite good sensitivity and specificity. A higher concordance rate between the PHQ-2 and PHQ-9 would translate into a greater advantage of the structured protocol approach for diagnostic evaluation among depressed clients, whereas a low concordance contributes to inefficiency and unwarranted burden on medical providers to perform depression evaluations on clients who are not clinically depressed. An alternative explanation to the diminished advantage of the structured protocol model is that the providers in the clinical acumen arm simply performed equally well as those in the structured protocol model with regards to correctly identifying clients who were clinically depressed and in need of a diagnostic evaluation and treatment. This has implications from a resource cost perspective, as the greater time burden created by the structured protocol is not cost-effective when the clinical acumen of trained providers performs well.

Among the clinically depressed, the slight advantage of the structured protocol model over clinical acumen with regard to prescription of antidepressant treatment was not statistically significant, likely for the same reasons described above to explain the lack of group differences with regard to diagnostic evaluation. In both models, nearly all clients who were diagnosed with depression by their provider were prescribed antidepressants, suggesting that any advantage of the structured protocol model lies in depression evaluation and diagnosis, not treatment uptake. The structured protocol model helps ensure that a client who screens positive is evaluated and diagnosed, but once a client is diagnosed there is little difference between the models. Diagnostic evaluation is a prerequisite for prescription of treatment; therefore, the degree of accuracy of the screening tool (which triggers diagnosis) influences the effectiveness, efficiency and cost of the structured protocol model relative to clinical acumen.

Depression care approaches that have used structured protocols have been shown to improve the quality of depression care delivered by non-mental health specialists relative to reliance on best practice guidelines and clinical acumen [12,14]; however, the clinical acumen model performed well in this study. We believe that the on-site, structured, ongoing supervision and mentorship from supervising psychiatrists, which was provided in both models, was crucial to this success, as suggested by published recommendations for mental health training programs [18]. However, it is important to note that the level of supervision provided in this study is much more structured and frequent compared to the type of supervision available as part of usual care in the Uganda health system; therefore, the structured protocol model would still be recommended for this setting if the high level of supervision provided in this study was not sustainable. We could have chosen a clinical acumen model that involved only depression care training to providers at the outset of the study, and follow-up supervision that was unstructured and only every few months, which would be more similar to the approach commonly used by Ministries of Health when trying to integrate new services into health care. This would have likely provided greater contrast to and evidence supporting the structured protocol model, but we believed the study would better inform policy makers by including a comparison model that represented a more viable model for successful implementation and sustainability of depression care.

More severe depressive symptomatology was associated with a greater likelihood of having had a diagnostic evaluation, and prescription of antidepressant therapy, which are indicative of quality depression care as clients with the greatest need were more likely to receive depression care. Better physical health functioning was also associated with receiving a diagnostic evaluation and treatment among those who were clinically depressed. This finding may seem contrary to the finding related to depression severity, as depression severity and physical functioning are often negatively correlated [32]; however, in the context of HIV disease, physical symptoms and functional impairment can resemble symptoms of HIV disease, and contribute to medical providers discounting physical symptoms as a manifestation of HIV rather than potential depression and therefore may be less likely to pursue depression evaluation [10].

This analysis focused on provider adoption of depression care delivery and the reach of depression care to clients at HIV clinics, but examined only early stages of the depression care process, from identification and diagnosis to prescription of treatment. A full implementation evaluation of these models must also include the quality or fidelity of treatment provided, including proper type and dose of the antidepressant prescribed, monitoring of side effects and symptoms, the retention of clients in care, whether clients successfully respond to treatment, and whether depression care is sustained over time once the research program ends. The implementation of this project will soon be completed, enabling us to analyze these latter stages in the depression care continuum.

There are other limitations to the study as well. The variation in rates of diagnosis and treatment across sites within each model of care suggests that there are factors unaccounted for in our analysis that contribute to implementation outcomes. Such factors could include aspects of the clinic environment and leadership, as well as structural aspects of the health care system that may vary across districts, and should be considered in future research [33]. The other key limitation is the small number of clinics in the study, which lowered the statistical power given the need to control for clustering effects. Some of the differences between the two depression care models appeared clinically meaningful (e.g., proportion of clinically depressed clients who received a diagnostic evaluation), but were not statistically significant. Unfortunately, the costs associated with including more study sites were prohibitive.

In conclusion, our findings provide evidence that trained lay workers and medical providers can successfully implement depression screening, diagnosis and treatment prescription when given training and ongoing supervision from mental health specialists. Compared to usual care practices within HIV clinics in Uganda, which is essentially void of depression care, both the structured protocol and clinical acumen models demonstrated a high level of adoption of depression care practices and reach of services as most clinically depressed clients were diagnosed and treated. The structured protocol model of depression care helps to ensure that depressed clients receive a diagnostic evaluation and treatment, but this advantage depends on how well providers using clinical acumen can accurately identify clinically depressed patients, which in turn is likely dependent on the strength of available supervision. These data lend further credence to the feasibility of non-mental health professionals to provide quality depression care when considered in conjunction with findings from other studies that have shown similar results in other regions of the world and with different patient populations [12–14]. Lack of mental health specialists should not be viewed as a valid reason for the relative absence of depression care in HIV and primary care settings in Uganda, the region of sub-Saharan Africa, and other low resource settings.

## Supporting Information

S1 File CONSORT extension for cluster trials checklist. (DOCX)

S2 File INDEPTH study protocol. (PDF)

S3 File Published INDEPTH study protocol manuscript. (PDF)
