# Supplementary material for: A Structured Protocol Model of Depression Care versus Clinical Acumen: A Cluster Randomized Trial of the Effects on Depression Screening, Diagnostic Evaluation, and Treatment Uptake in Ugandan HIV Clinics
Source: PLoS One. 2016 May 11;11(5):e0153132. doi: 10.1371/journal.pone.0153132 (PMC4864192; doi:10.1371/journal.pone.0153132)
Supplement: S2 File — (PDF) [file pone.0153132.s002.pdf]

Integration of Depression Treatment into HIV Care in Uganda (INDEPTH-Uganda)

Cluster Randomized Controlled Trial

Study Protocol

Funded by NIMH/PEPFAR

Principal Investigators:
Glenn Wagner (RAND)
Seggane Musisi (Makerere University)

TABLE OF CONTENTS

	Page
Study Overview	3
1.0 Introduction	4
2.0 Study objectives	5
3.0 Study design	5
4.0 Study setting	5
5.0 Selection and enrollment of participants	6
6.0 Interventions: Depression care models	7
7.0 Data sources	10
8.0 Measures	11
9.0 Statistical analyses	12
10.0 Study timeline	14
11.0 Human subjects considerations	14
Fig. 1: Depression and treatment eligibility assessment protocol	18
Fig. 2: Algorithm for dose or medication changes	18
Table 1: Components of the protocolized and clinical acumen models	19

STUDY OVERVIEW

DESIGN: A comparative trial that compares two implementation models for integrating antidepressant treatment into HIV care within 10 health care facilities in Uganda. Using a cluster randomization, 5 clinics are assigned to implement a protocolized model, and 5 others rely on the clinical acumen of trained providers. To evaluate the models, data will be collected from documentation mechanisms integrated into routine care, and from random samples of 100-150 patients enrolled at each site that screen positive for possible depression and are followed for 12 months. A cohort of providers will also be enrolled at each site and followed over 24 months. We will compare the two models on uptake of antidepressant treatment, and change in depression (treatment response), as well as other quality of care outcomes.

STUDY SITES: Of the 10 healthcare facilities participating in the study, eight are run by the Ministry of Health and two are private, faith-based, not-for-profit healthcare facilities; two are district hospitals and the others are designated as health centre III or IV facilities by the Uganda Ministry of Health, and are located in the districts of Mpigi, Mityana, Luweero, and Wakiso.

SAMPLE SIZE: 10 clusters (clinics), with 100-150 patients at each clinic.

POPULATION: Over the course of 12 months at each site, research coordinators (Masters level graduates with social science training) will recruit random samples of 100-150 patients who the triage personnel screen as positive for potential depression (PHQ-2 > 2), who are confirmed to be positive in the research coordinator's own PHQ-2 administration, and who are determined to be medically stable.

STRATIFICATION: To ensure the two study arms are balanced on size of clientele (small clinics serve 300-1000 clients, while larger clinics serve 1500-3000 clients), which could influence depression care processes, randomization will be conducted within pairs of clinics that were matched on this variable.

INTERVENTION: The trial is comparing two active task-shifting implementation models of depression care: a *protocolized model* in which care is provided largely by trained nurses who act as depression care managers, and a model that relies on the *clinical acumen* of trained primary care providers (most, but not all, of whom are nurses as well). An alternative approach to the use of a structured protocol to guide depression care by non-mental health professionals, the clinical acumen model (like the protocolized model) takes an active approach to depression care by integrating a brief routine depression screening process for all patients at each clinic visit, but what the primary care provider does with this screening information is up to there discretion as opposed to following a structured protocol.

PRIMARY OBJECTIVES: The objectives of INDEPTH-Uganda are to compare the two task-shifting models on (1) reach (screening and follow-up rates), adoption (treatment uptake), fidelity (quality of depression care implementation by the providers), and effectiveness (alleviation of depression symptoms) implementation parameters. A secondary objective is to assess the impact of depression treatment on key economic (work) and public health (ART adherence, condom use) outcomes.

IRB approval: The study protocol has been approved by Institutional Review Boards at RAND Corporation (protocol # 2012-0115), Mildmay Uganda, and the Uganda National Council for Science and Technology.

1.0 INTRODUCTION

In response to the NIH/PEPFAR RFA for implementation science and impact evaluation, this proposal addresses these priority areas: integration of HIV primary care and a common co-morbidity (depression), and examining depression treatment as a means to promote HIV care adherence and prevention of transmission. Sub-Saharan Africa (SSA) has undergone dramatic scale-up of HIV care and antiretroviral therapy (ART), with over 3 million on treatment in the region. The ultimate goal of HIV care is not only prolonged health and survival, but to provide a functioning level that enables clients to work, provide for their families, and prevent transmission of the virus to others. In fact, ART has led to dramatic reductions in mortality and morbidity, reduced infectiousness, and increased work productivity. However, our research in Uganda reveals depression to be a key threat to these critical public health benefits of HIV treatment. In our prospective cohort study that observed clients over the first year of care, multivariate analysis revealed depression to be the key impediment to ART adherence, whether or not someone was able to work, and consistent condom use.

As in other parts of the world, roughly half of all persons living with HIV/AIDS (PLHA) in SSA have elevated depressive symptoms, and 20-30% have clinical depression. Despite this high prevalence, and the public health consequences of depression, mental heath care is rarely integrated into HIV care programs, including those supported by PEPFAR; hence, depression is rarely diagnosed and treated. Antidepressants are effective in treating depression in PLHA, including in SSA as shown by our current research in Uganda, and inexpensive; however, the lack of trained mental health specialists is a major barrier to provision of treatment. Under current models of care, increased emphasis on depression treatment would fall in the hands of primary care physicians, and with most clinics having 1 or 2 physicians for seeing 100-200 patients daily, many depressed patients would likely still go undetected and bottlenecks of patients waiting for physicians would worsen. Task-shifting approaches in which nurses and other cadres take on tasks normally conducted by physicians have been used successfully in SSA in efforts to sustain ART scale-up, but not with regard to mental health treatment. Algorithm-based, nurse-driven management of antidepressant treatment has been shown to be effective with non-HIV patients in the U.S. and in developing countries, but has not been studied with PLHA or in SSA.

INDEPTH (***IN****tegration of* ***DEP****ression* ***T****reatment in* ***H****IV care*) ***Uganda*** is a cluster randomized trial that attempts to identify an effective, resource-efficient model for integrating depression treatment into HIV care in low resources settings such as Uganda. In the HIV clinics of 10 healthcare facilities, the trial is comparing two active task-shifting implementation models of depression care: a *protocolized model* in which care is provided largely by trained nurses who act as depression care managers, and a model that relies on the *clinical acumen* of trained primary care providers (most, but not all, of whom are nurses as well). An alternative approach to the use of a structured protocol to guide depression care by non-mental health professionals, the clinical acumen model (like the protocolized model) takes an active approach to depression care by integrating a brief routine depression screening process for all patients at each clinic visit, but what the primary care provider does with this screening information is up to there discretion as opposed to following a structured protocol. So both of these models are active task-shifting models of depression care, as the clinical acumen model goes beyond current usual care, which relies solely on primary care provider to assess and treat, or refer to external specialists—which has resulted in depression being severely under-diagnosed and treated.

2.0 STUDY OBJECTIVES

Primary Aims:
1. Compare the task-shifting (nurse) vs. standard (physician) models on the following quality of care indicators:
(a) depression treatment uptake, and (b) reduced depression (treatment response). *Hypothesis: Task-shifting model is associated with greater treatment uptake and depression reduction compared to standard model.*
2. Examine the relationship between change in depression and key economic and public health outcomes. *Hypothesis: Reduced depression is associated with better ART adherence, working, and more condom use.*

3.0 STUDY DESIGN

INDEPTH-Uganda is a comparative trial that compares two active task-shifting implementation models for integrating antidepressant treatment into HIV care within 10 health care facilities in Uganda. Using a cluster randomization, 5 clinics are assigned to implement a protocolized model, and 5 others rely on the clinical acumen of trained providers. To evaluate the models, data will be collected from documentation mechanisms integrated into routine care, and from random samples of 100-150 patients enrolled at each site that screen positive for possible depression and are followed for 12 months. We will compare the two models on uptake of antidepressant treatment, and change in depression (treatment response), as well as other quality of care outcomes.

4.0 STUDY SETTING

The study is being conducted in collaboration with Mildmay Uganda, a non-government organization that provides holistic outpatient HIV care at its own clinics, trains healthcare workers throughout Uganda and the region, and provides technical assistance in HIV care to healthcare facilities across Uganda. Of the 10 healthcare facilities participating in the study, eight are run by the Ministry of Health and two are private, faith-based, not-for-profit healthcare facilities; two are district hospitals and the others are designated as health centre III or IV facilities by the Uganda Ministry of Health, and are located in the districts of Mpigi, Mityana, Luweero, and Wakiso. Each facility is a hospital that operates an HIV clinic on specific designated days of the week, and it is in these clinics that depression care is being integrated as part of this study. The 6 larger clinics operate 2-3 days per week and generally have 1 clinical or medical officer and 3 to 5 nurses to provide primary HIV care to a clientele ranging from 1500-3000 clients and 80-120 patients are seen each clinic day. The 4 smaller clinics operate one day per week and are manned by 1 clinical or medical officer and 2-3 nurses; client base ranges from 350-1000, with 40-60 patients seen each day.

Consistent with task-shifting approaches becoming increasingly common in the context of ART scale-up across the region, at all sites nurses serve as primary care providers (along with the clinical/medical officers), and manage the prescription and monitoring of ART and other common HIV medications; more complex conditions or complications that arise are typically managed by the clinical/medical officer. All clinics have expert clients (volunteer experienced HIV clients who display exemplary HIV care adherence and are trained to provide peer support and assist in lower level tasks) and village health team (VHT) workers (community lay volunteers) who volunteer to take on tasks such as triage assessments, filing and retrieving charts, and keeping the clinic clean. No psychiatric or depression care services were being

provided at these clinics prior to the study; clients who developed significant psychiatric symptoms are referred to the nearest district or regional hospital for care.

5.0 SELECTION AND ENROLLMENT OF PARTICIPANTS

Over the course of 12 months at each site, research coordinators (Masters level graduates with social science training) will recruit random samples of 100-150 patients who the triage personnel screen as positive for potential depression (PHQ-2 > 2), who are confirmed to be positive in the research coordinator's own PHQ-2 administration, and who are determined to be medically stable.

A cohort of providers from each site will also be enrolled and followed over the 24-month implementation to examine change in knowledge, attitudes and practices regarding depression care and perceived impact of the implementation on clients, providers and clinic operations.

All patient and provider participants will provide written informed consent prior to enrollment.

5.1 Recruitment and enrollment process:

The consent forms and IRB protocol for the study will be submitted for review and approval by the RAND IRB, and the IRB used by Mildmay (Makerere University, Faculty of Medicine). Once approval is obtained from these IRBs, the protocol will be sent to the Uganda National Council of Science and Technology for final approval (this typically takes no longer than one month).

HIV care providers

We will approach clinic staff (DCM nurses, primary care providers) at the participating sites to participate in the study by providing self-report data on their experience with the depression treatment models. Written informed consent will be obtained. It will be made clear that their participation is voluntary and that there will be no ramifications related to their job, job evaluation, or otherwise if they decide not to participate in the research data component.

HIV patients

Patients who have screened positive for depression on the routinely administered PHQ-2 will be informed of the study by one of their providers (nurse or doctor). If interested in participating in the study the patient will be referred to the site coordinator for a detailed description of the study, confirmation of eligibility and consent procedures. It will be made clear to patients that their decision of whether to participate or not will have no impact on their clinical care or ability to receive depression treatment if needed. The site coordinator will describe the details of the study, including the nature of the patient's involvement in the study, the possible risks and benefits of participation, and the patient's ability to withdraw from the study at any time without consequence. The patient will have the opportunity to ask questions about the study, after which informed consent will be obtained and documented by having the patient read and sign a consent form, which will be stored in a locked file cabinet. Each participant will be given a copy of the consent form to keep. No modifications or waivers of the elements of consent or the requirement for documentation of consent have been requested from our Institutional Review Board.

5.2 Participant tracking procedures Contact information (address, phone numbers) will be collected at enrollment and verified at follow-up visits. We will collect contact info of two people who are likely to know how to reach the participant if needed, and will ask participants what to say, and avoid saying, when contacting them.

6.0 INTERVENTION: Task-Shifting Depression Care Models

6.1 Protocolized Model

Drawing from collaborative care models of depression treatment such as Partners in Care, MANAS and STAR*D, the protocolized model implements an algorithm-based, nurse-driven approach to administering depression diagnosis and antidepressant therapy. While other collaborative care models incorporate psychotherapy and reserve antidepressants for severe depression, our model focuses solely on the use of antidepressants. We believe that use of medication requires relatively less trained resources and personnel time than would be needed to add a form of psychotherapy, resulting in a more reliable and scalable treatment for resource constrained settings. The components of the protocolized model are outlined below and summarized (in comparison with the clinical acumen model) in Table 1.

Routine depression screening: All adult clinic patients will be screened for depression at each clinic visit using the first two items of the Patient Health Questionnaire (PHQ-2), administered at the triage station (along with measurements of body weight, blood pressure, pulse and elicitation of presenting health problems) by expert clients or VHT workers. The PHQ-2 assesses depressed mood and loss of interest; patients who screen positive for depression (3+ scores; range: 0-6) will be referred to the nurse in the protocolized model for further depression evaluation if medically stable; in the clinical acumen model, PHQ-2 data are relayed to the primary care provider. Medical stability is defined as not about to start (or recently started) ART or treatment for an acute opportunistic infection (see Figure 1). Further evaluation is deferred for medically unstable patients until their condition and treatment is stable, as mood may improve once they are medically stable, and starting antidepressants simultaneously with other medical treatments would complicate side effect and response evaluation. However, depressed patients who are medically unstable will be evaluated for suicide risk and the need for immediate treatment.

Depression diagnosis: Further evaluation of patients who screen positive for depression and are medically stable will be conducted using the full 9-item Patient Health Questionnaire (PHQ-9). Patients who score greater than 9 on the PHQ-9 (range: 0 to 27), which has been shown to correspond highly with Major Depression as determined by a diagnostic interview, will be further assessed using the criteria for Major Depression on the Mini Neuropsychiatric Interview (MINI; 5 symptoms scored as 3 on the PHQ-9, at least one of which is depressed mood or loss of interest). Providers are trained to further assess antidepressant eligibility using the MINI screening items for bipolar disorder, psychosis and substance abuse, as well as medical contraindications (e.g., pregnancy or breast feeding, seizure disorder); for patients with these conditions, the supervising psychiatrist will be consulted to determine appropriate treatment.

Prescription of antidepressants: If eligibility for antidepressant therapy is confirmed, the provider (nurse in the protocolized model and primary care provider in the clinical acumen model) will educate the patient about depression as a disease, and antidepressant treatment, including: prognosis, the goal of symptom remission, that treatment often takes 2-3 weeks to have an effect, and that side effects are generally minor and temporary. Helping the patient to understand the treatment is intended to encourage adherence to treatment. The patient will then receive a prescription and supply of either fluoxetine or imipramine prior to departing the clinic, which the provider will select based on the patient's presenting symptoms and psychiatric history; fluoxetine will be prescribed unless the patient presents with insomnia, sexual dysfunction or history of Bipolar disorder. Fluoextine has been shown to have some drug interactions with HIV protease inhibitors, but protease inhibitors are rarely used in Uganda.

Monitoring of treatment response and side effects: After treatment prescription, the patient will return to see the prescribing provider two weeks later for monitoring of side effects, treatment response and any need for change in dosage or medication. The visit schedule will then become monthly until the patient has been in remission (defined as PHQ-9 < 5 and tolerating any side effects) for one month, at which time the visit schedule will be modified to match the patient's HIV care visit schedule (typically every 2-3 months), but no less than every 3 months; if the patient experiences a relapse, visits will return to monthly until in remission. At each visit, the provider will assess depressive symptoms (using the PHQ-9), presence of side effects and strategies for managing such symptoms, as well as enquire about medication adherence. Psychoeducation regarding depression treatment will continue and be emphasized as needed. A Depression Treatment Registry will been instituted at each site in which the above listed parameters are recorded for each visit, as well as medication and dosage prescribed. These data will be reviewed during supervision and used for reference in future follow-up visits, as well as for tracking fidelity to the treatment protocol.

Starting daily dose is 20 mg for fluoxetine and 50 mg for imipramine; imipramine will increase to 75 mg after one week. Patients remain on this low dose until their second follow-up visit (Week 6), at which time the determination of the need for a change in dose or medication will be considered based on measures of depressive symptoms and side effects (see Figure 2). *For patients tolerating any side effects*: dose will remain the same if they are fully responsive to treatment; for partial responders, the provider will decide whether to maintain the dose or increase by one increment (add 20 mg of fluoxetine or 25 mg of imipramine); nonresponders will have their dose increased by one increment. *For patients not tolerating side effects*: if a full or partial response to treatment, the current dose may be maintained and side effects addressed (with pharmacologic or other types of strategies) or the provider may reduce the dose or change the antidepressant; for nonresponders, the antidepressant will be changed.

This algorithm-based treatment decision process is repeated at each monthly visit until the patient is fully responding and in remission-- the medication dose will then remain the same (unless subsequent changes are needed due to side effects or relapse). Maximum dose is 80 mg/day for fluoxetine and 300 mg/day for imipramine. If patients don't respond to or tolerate fluoxetine or imipramine, amitriptyline is an option; alternatively, a medication that is being partially responded to may be continued in hopes that response will improve over time. Concomitant medications for associated symptoms such as sleep disturbance, anxiety or agitation, or side effects (e.g., sexual dysfunction), may be used and will be documented.

Treatment discontinuation: Once the patient has been in remission for at least 6 months, treatment will be discontinued, unless the patient has a history of multiple episodes of Major Depression, in which case treatment will be maintained for two years. If on treatment at end of study, treatment will be maintained as part of usual care. Drug supply is purchased by the study to ensure no drug stock outs, but both medications are available free of charge to the clinics through the Ministry of Health.

6.2 Clinical Acumen Model

Although psychiatric treatment is generally not currently available in HIV clinics (nor general primary care clinics) in Uganda, clinics that are looking to increase their provision of depression treatment are likely to follow the model used in many other parts of the world, which is to train primary care providers to identify and treat depression. Similarly, the clinical acumen model of depression care relies on the discretion of primary care providers who receive depression care training to provide depression diagnosis and treatment as deemed warranted. To facilitate this

process, like the protocolized model, the clinical acumen arm also includes routine screening of all adult clients and monthly on-site supervision from study psychiatrists. However, this model relies on the clinical judgment of the primary care provider to decide whether to further evaluate and treat patients who screen positive for possible depression, as opposed to a structured protocol (see Table 1 for an outline of the model and how it compares to the protocolized model). Another difference between the models is that depression care is managed entirely by nurses (with oversight from the clinical/medical officer in charge) in the protocolized model, whereas in the clinical acumen model all primary care providers (who are comprised of nurses and clinical/medical officers) are expected to provide depression care.

6.3 Training, Supervision and Monitoring

Training and ongoing supervision are critical for ensuring quality of care and fidelity to the depression care models, and to provide technical and emotional support to the providers. With management of antidepressant treatment being new to the clinic staff, the supervision is particularly important and will help to increase the confidence of the providers in their ability to provide high quality care. Supervision is also an opportunity for the providers to refresh and upgrade their new skills in an ongoing forum that reinforces the value of the investment they have made in the training and the integration of depression care into their HIV care practice. Furthermore, working with patients who are depressed can be stressful and emotionally draining, so the support from supervision can help prevent burnout and job turnover. The training and supervision provided to the clinic staff in both arms of the study is very similar; the only differences are that the providers in the clinical acumen arm will not be trained to follow the parameters of a structured protocol when further assessing patients who have screened positive for possible depression. The manual used to train the providers, and which was provided to each provider to take back to their clinic for reference, is included as an attachment.

Start-up Training: Training will start with the study's lead investigators and psychiatrists conducting an intensive one-day training workshop with the clinic staff (expert clients/VHTs, nurses, clinical/medical officers) to train them on the structure and goals of the study, and each component of the treatment model. One workshop will be held for the clinics in the protocolized arm, and a separate workshop for those in the clinical acumen arm. All nurses and clinical/medical officers at each site will be trained so that duties can be evenly spread, to build overall capacity for depression care, and to mitigate against the effects of occasional staff transfers. Training will consist of didactic instruction, interactive role-playing, and small breakout group discussions. Following the workshop, tools to facilitate implementation of the model (laminated copies of the PHQ-2 for the triage station, and PHQ-9/MINI for each nurse/prescribing provider, Depression Treatment Registry, psychoeducation posters and flipcharts) will be delivered to each site, and supervising psychiatrists will be on-site one day a week for 4-6 weeks (until both the supervisor and nurse are comfortable with the nurse's competency in implementing the protocol) to provide training and mentorship (e.g., sitting in on and co-conducting assessments), after which ongoing on-site supervision will become monthly.

Ongoing Supervision: Supervision will be conducted in one-on-one sessions between the site supervisor and each nurse (protocolized arm) and primary care provider (clinical acumen arm), as well as group meetings with all clinic staff involved with implementation of depression care at the site. Furthermore, the supervisor will be available 24/7 for emergency or suicide crisis consultations. During individual supervision, the providers will present new treatment cases since last supervision and problematic or nonresponding cases, allowing the supervisor to discuss the patient's presenting symptoms and the treatment plan for the patient, including side effect management and dose change recommendations. Clinical notes from the Depression

Treatment Registry will be used in the case reviews. Goals of treatment for the individual patient will be discussed and recorded for review at subsequent supervision sessions. The group meetings will provide an opportunity for the clinic staff to work as a team to trouble-shoot any challenges that arise, share experiences and provide peer support to each other in managing depressed patients, which can be taxing emotionally as well as fulfilling.

Monitoring Fidelity: Ongoing supervision and review of cases is our primary mechanism for monitoring the fidelity of the implementation of the treatment models. At each monthly supervision, supervisors review the charts of all patients prescribed antidepressants within the past month, as well as 10 randomly selected charts of patients receiving ongoing antidepressant treatment monitoring. The charts will be reviewed for whether diagnosis, symptoms and side effect assessment, and dosing were appropriately performed, and if the patient returned for follow-up visits. Results will be aggregated and monitored on a monthly basis, as well as used to inform the target areas for supervision.

7.0 DATA SOURCES

To evaluate the two task-shifting models, we will compare the implementation, effectiveness, and cost-effectiveness of these models over a 24-month period. Using the RE-AIM framework, we will evaluate the *reach* of depression screening and treatment, *adoption* of depression treatment knowledge and intervention by providers, *quality of implementation* (fidelity to depression care model), and *effectiveness* of depression treatment on patient outcomes. As outlined in Table 2, these implementation domains and corresponding outcomes will be assessed from data triangulated from these data sources: (1) data abstracted from data collection mechanisms integrated into routine care; (2) monthly logs summarizing chart abstracted data completed by the supervising psychiatrists; (3) survey data from a longitudinal cohort of randomly selected patients at each site who screen positive for potential depression (PHQ-9 > 2); and (4) survey data from longitudinal cohort of providers involved in provision of depression care at each site. Survey data from the patient cohort will also be used to assess how change in depression is related to key economic and public health outcomes. These data sources are described in more detail below.

Routine data abstraction: We will assess the implementation of screening (% of adults screened; % screened who screen positive) by abstracting data from the Triage Book, which the triage station at each site uses to list the clients who attend each day of the clinic, their vital signs, and PHQ-2 score. PHQ-9 and MINI administration data will be recorded in the patient's clinic chart. Depression Treatment Registry books will be installed at each site for providers to document clients who have started antidepressant therapy and their depression diagnosis, and records of each follow-up visit that include PHQ-9 score, presence of side effects and medication and dose prescribed.

Supervision log data: From the chart reviews that the supervisors conduct each month at each site, monthly aggregate scores are recorded for newly prescribed patients (number with correct diagnosis, correct prescribed antidepressant, and correct prescribed dosage) and patients in ongoing treatment (number correctly assessed for depressive symptoms and side effects; number correctly dosed; and number who returned for scheduled follow-up visit in past month).

Longitudinal client cohort: The client research participants will be assessed at baseline and months 6 and 12. The surveys will be administered by the research coordinator using computer assisted personal interview technology, and will include the PHQ-9 (to independently assess the presence of depression) as well as measures of demographic and background characteristics,

work activity, clinical appointment and ART adherence, quality of life, sexual behavior and measures of psychosocial functioning. All measures have been used successfully in our prior research in Uganda and have been translated into Luganda, the primary native language used in the study setting. Participants will receive 10,000 Ush (~$4 USD) for each assessment.

Longitudinal provider cohort: The provider cohort will be surveyed at the start of intervention implementation and then at months 6, 12 and 24. The measures will include perceived competency in providing depression care, quality of depression treatment provided, effects of the treatment model on clinic functioning, provider burden and patient flow, and impact of depression treatment on the patient's quality of life.

8.0 MEASURES

Quality of depression care measures

Depression is assessed with depression module of the Patient Health Questionnaire. The 9 items are the 9 DSM symptom criteria for Major Depression; a "past 2 weeks" time frame is used and each item is scored from 0 'never' to 3 'every day'. Total score ranges from 0-27; scores 1-4 is min. depression, 5-9 'mild', 10-14 'moderate', 15-19 'moderately severe', and 20+ 'severe'. The first two items (depressed mood and loss of interest) compose the PHQ-2 screen, and a sum score $\geq$3 represents a positive screen for depression. The PHQ-2 has high specificity (95%) and sensitivity (85%) for PHQ-9 diagnosed depression in PLHA in SSA, and PHQ-9 has high sensitivity and specificity (both 88%) with MDD by diagnostic interview. The Mini Neuropsychiatric Interview screening modules will be used to rule out Bipolar Disorder and psychosis.

Response to antidepressant treatment: full response defined as PHQ-9 < 5 and tolerating any side effects.

Antidepressant side effects will be assessed with an adapted Antidepressant Side Effect Checklist (ASEC), which enquires about symptom severity of common antidepressant side effects (e.g., dry mouth, nausea, diarrhea) on a scale of 0 'absent' to 3 'severe' and perception of the symptom as a side effect of the antidepressant. It will be used by the DCM in the task-shifting model, as well as the survey assessment.

Quality of life and functioning will be assessed with the Medical Outcomes Study HIV Health Survey. A Luganda version has been validated. The MOS-HIV includes subscales of physical function, social and role function, cognitive function, pain, mental health, energy, distress about health, quality of life and overall health.

Public health outcomes

Work We will draw on the employment module of the Uganda National Integrated Household Survey to assess work activities (e.g., formal salary, farming, to selling goods) and amount of income and hours worked.

Sexual risk behavior will be assessed including frequency of sexual intercourse; number and type (main, casual, commercial) of sex partners, and condom use (at last sex and in general in separate items) over past 6 months, as well as HIV status disclosure from and with reported sex partners.

ART and antidepressant adherence. We will ask about number of missed doses over past 7 days, as well as a visual analog scale to measure adherence (0 to 100%) to ART (if applicable) and antidepressants. All patients receive their medication from the clinic pharmacy, so pharmacy refill data will also be used to assess a composite measure of refill rate across ART medications, as well as the antidepressant. **Clinic attendance** will be gauged from chart abstraction and self-report about missed appointments in past 6 months.

Other measures
Demographics will include age, sex, ethnic tribe, position in household, education level, relationship status. ***Alcohol use*** will be assessed using the 4-item Alcohol Use Disorders Identification Test (AUDIT).
HIV characteristics: HIV test date, WHO disease stage, CD4, and medications used will be chart abstracted.
Treatment-related attitudes and beliefs. Measures of perceived efficacy of treatment (2 items), perceived social support for treatment (2 items), and adherence self-efficacy (1 item), adapted from ACTG measures.
General self-efficacy and expected outcomes. We developed 5-item Likert-scale measures of self-efficacy and expected outcomes for tasks such as work, condom use, caregiving, and forming social relationships.
Use of mental health services (e.g., psychosocial counseling) over past 6 months will be assessed.

Process Measures: We will collect clinic structural data and implementation process measures in order to identify lessons learned for scaling up depression treatment and inform the generalizability of our findings.
Structural measures: Site visits and interviews with clinic administrators will be conducted prior to the implementation of the treatment models and then every 6 months for 24 months to assess: staff composition (number and type of providers), staff turnover, number of patients in care, presence of ancillary services (e.g., nutrition, school fees support, income generating activities), change in donor/funding levels, availability of ART.
Provider experience: We will develop Likert rating scales for DCMs and physicians to complete on a quarterly basis from the start of treatment implementation to assess their perception of: self-efficacy in providing depression treatment; quality of depression treatment provided; effects of the treatment model on clinic functioning, provider burden and patient flow; and impact of depression treatment on the patient's quality of life.
Patient experience: Patients enrolled in the study will be asked to rate their satisfaction with the treatment effects on quality of life and perceived competency of providers to manage treatment at each follow-up visit.
Patient attendance: Patient attendance at antidepressant monitoring visits will be recorded.

9.0 STATISTICAL ANALYSES

9.1 Power analysis for primary aims. With 10 clusters, this study is powered to detect medium to large effect sizes; nonetheless, the results will be among the first of their kind, hence informative for subsequent analysis and policy. We calculated detectable differences for different intracluster correlation coefficient (ICC) values. Data from developing countries indicate low correlations of health outcome variables within primary care settings (improving power), including .03 for depression in a collaborative care trial in India, and .011 for maternal/perinatal health outcomes in 8 Latin American countries. ICC tends to be higher for process related measures (which would include the focus of Aim 1a): median=.16 for the same 8 country study.

Aim 1a (treatment uptake): With 150 patients per site and assuming 30% antidepressant uptake in the standard model and ICC=.16, we will be able to detect an increase of 28% in uptake rates between the task-shifting and standard clinics (α =.05, 80% power; one sided test). For ICC=.25 the increase is 34%. Given the systematic procedural difference in the two arms, we expect the actual difference may exceed these amounts.
Aim 1b (depression change): For measuring change in PHQ-9 in an intention to treat (ITT) analysis using the whole sample, with ICC =.025, s.d. =3.9 (from our current study), and 10% attrition, we'll have power to detect a 1.26 difference on the PHQ-9 (α=.05, 80% power; 2-sided

test); for ICC=.01 and .05, detectable differences are .92 and 1.71. For analysis of those receiving antidepressants, we estimate 30% (n=45) of the standard arm patients will be treated (a larger share is expected in the task-shifting arm, which will improve power). With ICCs =.01, .025, and .05 (s.d. = 3.0), and 10% attrition, we will be able to detect changes of 1.00, 1.20, and 1.46 in the PHQ-9. These correspond to medium effect sizes (Cohen's *d*) of 0.33, 0.40 and 0.49.

9.2 Statistical Analysis

The analyses must account for clustering of the data, unless the only comparisons are of cluster means or proportions, using clinic as the unit of analysis. For most outcomes we will perform such an analysis first, using standard t-tests or, where the observations per clinic vary, t-tests weighted by cluster-size. However, relative to individual level analysis this approach is limited by low power (low number of clusters); inflexibility in adjusting for baseline individual level covariates; and inability to model direct impacts of patient characteristics and their interactions with treatment. Therefore we will use regression methods on individual level data for most analyses, and the discussion below is largely restricted to these approaches. While such methods can directly model correlations among units in a cluster (random effects or multilevel models) or more simply adjust standard errors for clustering (GEE, robust standard errors), these approaches are not reliable when number of clusters is small: reliably estimating the ICC to adjust standard errors typically requires 20 or more clusters. Hence, we'll use standard regression methods, but rather than attempt to estimate ICC directly, we will explore the sensitivity of significance levels and conclusions to a range of plausible ICCs values for the outcomes.

<u>AIMs 1a: Depression Treatment Uptake</u>: We'll use logistic regression to compare the proportions of depressed patients (according to PHQ-2) receiving depression treatment in task-shifting vs. control arms. We expect that most patients whose PHQ-2 screen is followed up with a formal evaluation and diagnosis of depression will be prescribed antidepressants, so we are not examining this variable separately from treatment uptake; also, it would be difficult to ascertain if the doctor performed a formal evaluation in the standard arm. The treatment uptake outcome will be estimated using covariates to adjust for baseline characteristics such as age, sex, and physical health. We will add interactions of these covariates with study arm to assess which types of patients are likely to be diagnosed and treated in the task-shifting vs. standard models. For example, it is possible that nurses, who are largely female, are more likely to recognize and treat depression in female clients.

<u>AIM 1b: Change in Depression (treatment response):</u> The effect of task-shifting on treatment response (change in PHQ-9) will be examined using an ITT approach and the whole sample. We will use a repeated-measures, mixed-model approach allowing for correlation of errors over time for an individual (for which the sample size of our individual level data is adequate). The model has the following form:

$$(1)\ \text{Depression}_{it} = \alpha + \beta(\text{treatment model})_i + \gamma(t) + \delta(\text{treatment model})_i(t) + \theta_i + \varepsilon_{it}$$

Where *t* is time period, β controls for baseline differences between the two, γ is the trend in the outcome common to both groups, and δ is the interaction of treatment model with time and shows the intervention effect (task-shifting relative to the control). θ_i is an individual random effect and ε_{it} idiosyncratic period specific error. A vector of individual level covariates X_i will be added to estimate the effects of patient characteristics and interactions of X_i and task-shifting will be used to test for moderators: e.g., do effects of the task shifting model vary by the patient's physical health at baseline? As this is ITT, the estimates will capture the average benefit for depressed PLHA, including those who do and do not receive antidepressant treatment.

To assess effects on those receiving antidepressants, we will estimate (1) on the sample of those treated in both arms; however, randomization does not ensure equivalence of the two groups, since the process of selection into diagnosis and treatment will likely differ. In particular we might expect more seriously depressed patients to be treated in the standard arm. This can be addressed by including baseline PHQ-9 in the regression, or with fewer parametric assumptions, using propensity score matching to match similar patients in control and treatment arms. As both approaches assume that observables fully control for differences across arms in relevant characteristics of treated patients, more caution will be needed in interpretation of the results.

AIM 2: Assess relationship of depression and public health and economic outcomes: We will first examine correlates of baseline depression (PHQ-9) including ART adherence, condom use, and work activity. Multi-variate analysis will take advantage of repeated observations per individual to model the effect of changes in depression on changes in these outcomes (individual fixed effects), controlling for unmeasured individual characteristics that may affect both depression and the outcomes. However, it does not control for simultaneity (e.g., change in work status leads to lower depression). With 3 time-points, instrumental variables approaches combined with fixed effects potentially can deal with this problem, using baseline depression to predict change in depression from month 6 to 12, if baseline depression is strongly correlated with change in depression over time. The fixed effects model will also indicate important time patterns in depression and the other outcomes.

10.0 STUDY TIMELINE

Study Activity	Year 1				Year 2				Year 3			
IRB approvals; training of study personnel	X	X										
Implementation of depression treatment models			X	X	X	X	X	X	X	X		
Prospective cohort data collection			X	X	X	X	X	X	X	X		
Statistical analysis and write-up							X	X	X	X	X	X

11.0 HUMAN SUBJECTS CONSIDERATIONS

11.1 Potential Risks to Participants

Side effects and interaction effects of antidepressant therapy

The standard antidepressants to be used in this study include primarily fluoxetine and imipramine; amitriptyline is also available in Uganda but seldom used. The side effect profiles of these agents are thought to be relatively mild and infrequent, with the most common side effects including nausea, dry mouth, insomnia, headache, sexual side effects, somnolence, dizziness, sweating. These adverse events often dissipate as treatment continues, are reversible with treatment discontinuation, and rarely cause patients to decide to terminate therapy. It is also possible for antidepressants to have negative interaction effects with other medications being taken, including HIV antiretrovirals and anti-TB drugs, which can be manifested through physical symptoms and/or measures of immunosuppression (e.g., CD4). However, while interactions between these drugs are always conceptually possible, for the most part there is little evidence of significant interactions effects between antidepressants and HIV antiretrovirals. There is some data suggesting interaction effects between fluoxetine and HIV protease inhibitors, but very few patients are prescribed protease inhibitors at the study sites because NNRTI-based ART regimens (which do not contain a protease inhibitor) are the first line regimens in usual HIV care practice in Uganda and most of SSA. If drug interactions become evident, the primary care provider will consider dose modifications of the antidepressant or change in antidepressant.

Psychological distress
The participant may experience psychological distress while talking about issues raised during assessment interviews. A patient's depression could worsen, but this would not be expected to be a result of study participation.

Confidentiality
There is the potential for violation of participant confidentiality, but we believe the safeguards that we have integrated into the study methodology, as described in section E.5, will effectively limit these risks.

11.2 Protection Against Risk

Side effects and interaction effects of antidepressant therapy. If a participant receives antidepressant treatment, they will first receive a medical screen and psychiatric evaluation for appropriateness of antidepressant therapy as part of the depression treatment models prior to prescription of treatment by the primary care provider. At the sites implementing the task-shifting depression treatment model, the treatment response and side effects will be monitored by the DCM with a frequent visit schedule (treatment baseline, week 2, week 4, and then monthly thereafter until response, followed by every 3 months); in the standard, physician driven model, monitoring will be dependent on the primary care provider (who will receive training on use and monitoring of antidepressant therapy) as part of usual care. Patients will have 24-hour access to their primary care provider via phone for emergency contact. When side effects are reported, decisions related to dose reduction or discontinuation of treatment will be determined by the DCM and primary care provider, as outlined in the treatment model protocol. To monitor for possible interaction effects with HIV antiretrovirals, physical and adverse symptoms that are suspected to be possible interaction effects will be discussed between the treating providers and the site's supervising psychiatrist in their regular, ongoing supervision; also, CD4 count (and viral load, though it is rarely available) will also be monitored for potential interaction effects.

Psychological Distress. Participants have the right to refuse to answer specific interview questions and can stop the interview at any time. If the patient's depression worsens and they are on antidepressants, changes to the medication dose or new antidepressant will be considered as indicated by the protocol, and the supervising psychiatrist will be consulted if needed. Some of the clinic sites have counselors, which could be asked to provide some supportive psychosocial counseling if desired by the patient.

Confidentiality. To protect confidentiality, the following steps will be taken: All research data will be kept in locked file cabinets and will be available only to research staff directly involved in this project. Data will be identifiable only by study numbers and patient initials. HIV status will not appear along with any personal identifying information. The hand-held computer devices used to administer the computer-assisted personal interviews (at baseline and Months 6 and 12) will not collect any identifying information; only the study ID number and patient initials will be entered into these devices. The data on these hand-held computers will be password-protected and data will be uploaded to a study desktop computer on a daily basis. Personal information including subject's name, address, and phone number will be entered on a tracking form, stored separately from all research data, and kept in a locked file cabinet at the study site. Research interview data collected from the participants will be kept confidential and not be shared with the patient's physician or other clinic staff; the exception is the depression and psychiatric measures (PHQ-9, MINI, side effect measure), which will be shared with the patient's providers in order to optimize depression treatment. Patients will be informed of these conditions during the informed consent process.

11.3 Potential Benefits to Study Participants
All patient participants will be compensated 10,000 Ush ($5 USD) for completing each primary research interview (baseline and Months 6 and 12); this is the standard amount used in Uganda for this type of interview and is recommended by the Uganda National Council for Science and Technology. Participants will not be compensated for visits that are just for depression treatment management.

11.4 Importance of the Knowledge to Be Gained
If we demonstrate that the task-shifting approach to depression treatment is feasible, effective and well incorporated into clinic systems, it will establish a model that addresses the human resource challenges to building the capacity for sustainable depression treatment. Findings will have implications not only for PLHA in SSA, but also other developing regions, non-HIV populations, and treatment of other diseases.

11.5 Data and Safety Monitoring Plan

Potential Adverse Events. We do not anticipate any medication-related adverse events beyond that of routine HIV and ART medical care, and use of antidepressant therapy. Patients who are treated with antidepressants will have been diagnosed with major depressive disorder and treated with standard antidepressant therapy. Only FDA approved antidepressant medications will be used. All patients will have been cleared medically for initiation of antidepressant therapy by their primary care provider. All patients will be assessed and monitored with regards to psychiatric symptoms and treatment side effects (including suicidality and potential interaction effects with ART) by the DCM on a frequent standardized schedule if at a site implementing the task-shifting treatment model, or as consistent with usual care at the sites implementing the standard physician driven treatment model. Antidepressant side effects are alleviated by dose reduction, and discontinuation if needed. Antidepressant side effects are reversible, and therefore terminate when the medication is discontinued.

Protocol for suicidal ideation: With the study population being depressed, some will express suicidal thoughts during the PHQ-9 assessment of depressive symptoms. In our current study, 27% of depressed patients expressed any suicidal ideation (14% had frequent thoughts) at treatment baseline. The DCM and physicians will be trained to implement the following protocol when patients report suicidal thoughts: an assessment will be made of the severity of the ideation, intent and means for carrying out any intent for suicide; this will then be followed with the activation of a plan to keep the patient safe, which could vary from more frequent contacts/visits, to establishing a suicide contract that includes 24-hour supervision of the patient by a community member, to inpatient admission for psychiatric care.

Potential interaction effects between antidepressants and ART will also be monitored in terms of both physical adverse events and also adverse changes in immunosuppression (e.g., CD4).

Participants whose psychiatric status significantly deteriorates, or who becomes a suicide risk, and who are in need of psychiatric hospitalization will be admitted to Mulago Hospital's Mental Health Unit. The supervising psychiatrists all have direct admitting privileges to Mulago and other hospitals located closer to the study sites, which lends itself well to affecting immediate psychiatric hospitalization and treatment to participants found to be suicidal or at risk for harming themselves or others.

In addition, a single, independent, clinical specialist in the treatment of depression (a psychiatrist from Makerere/Mulago Hospital) will serve as an independent monitor for the study. To allow effective monitoring, the independent monitor will be provided with periodic reports which include subject enrollment, subject retention, the number of patients who drop out of the study with reasons for dropping out, and a listing of all adverse events (AEs) that are plausibly

related to antidepressant therapy or other study procedures. Periodic reports will be provided to the independent monitor at six month intervals; however, AEs that are considered directly related to antidepressant therapy or other aspect of study participation will be reported immediately to the monitor, the IRBs, and NIH. After review of the periodic reports, the independent monitor may ask for clarification or additional information from the PI. After such information is provided, if requested, the independent monitor will make a recommendation regarding the continuation, modification, or termination of the study. All communications from the independent monitor will be shared with the IRBs and NIH.

Data Integrity. Identifying information including subject's name, address, and phone number will be entered on a tracking form, stored separately from all research data, and kept in a locked file cabinet at the study site. The hand-held computer devices used to administer the computer-assisted personal interviews (at baseline and Months 6 and 12) will not collect any identifying information; only the study ID number and patient initials will be entered into these devices. The data on these hand-held computers will be password-protected and data will be uploaded to a study desktop computer on a daily basis. All paper files are stored in locked file cabinets, and electronic files are stored in password-protected files. Furthermore, both paper and electronic files will be identified only by a participant's ID number. Identifying information linking participants to their study ID number will be retained in a locked cabinet.

Figure 1: Depression and Treatment Eligibility Assessment Protocol

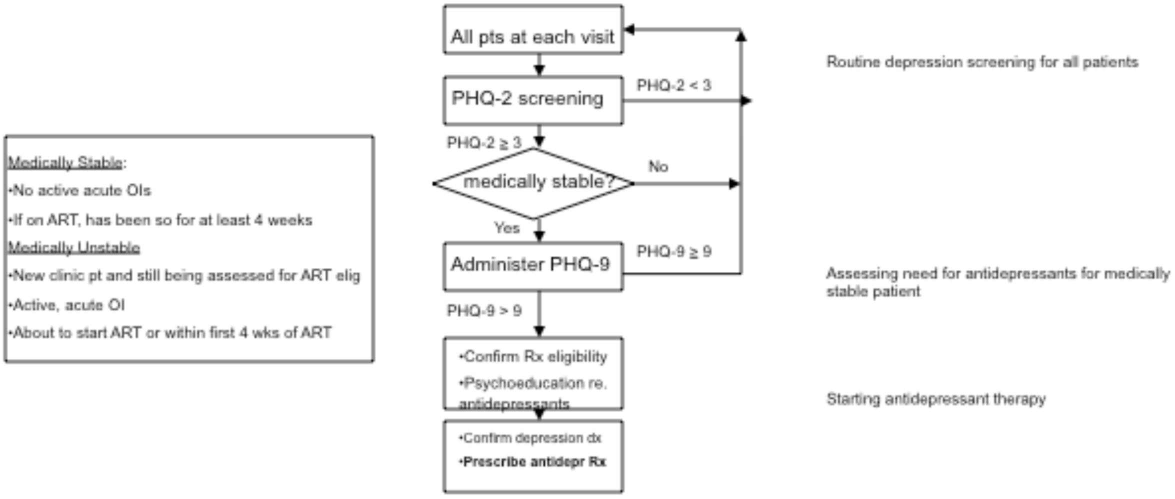

Figure 2: Algorithm for Dose or Medication Changes

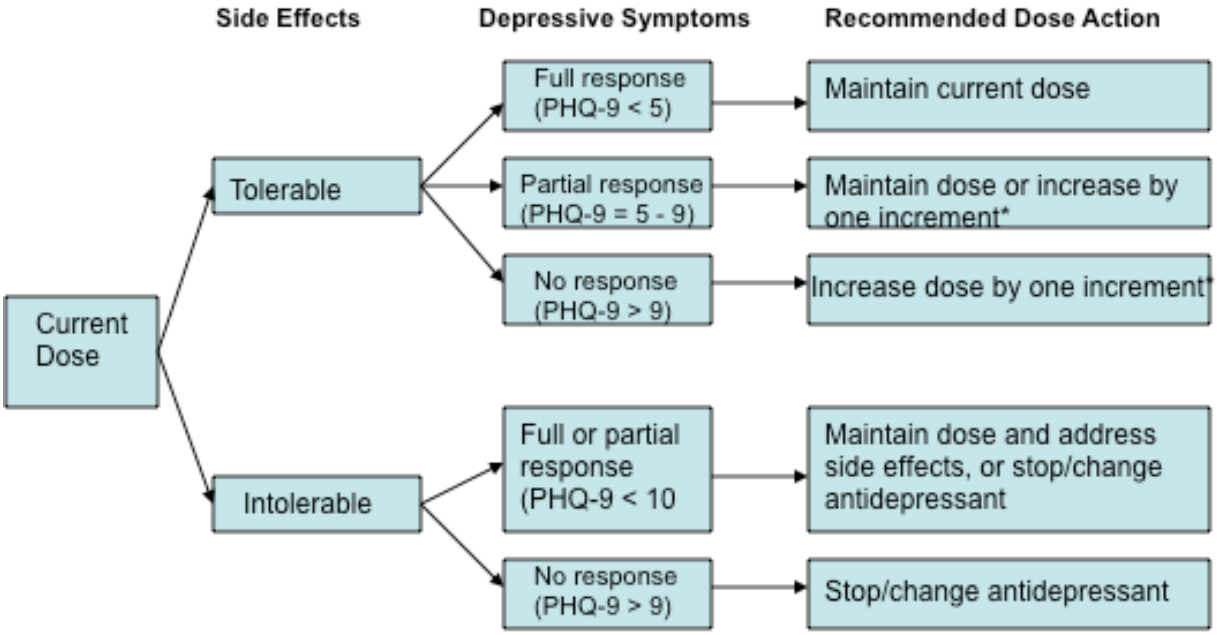

* One increment: fluoxetine = 20 mg/day; imipramine = 50 mg/day
